# Leonurine: a comprehensive review of pharmacokinetics, pharmacodynamics, and toxicology

**DOI:** 10.3389/fphar.2024.1428406

**Published:** 2024-07-19

**Authors:** Siyu Liu, Chen Sun, Hailin Tang, Cheng Peng, Fu Peng

**Affiliations:** ^1^ State Key Laboratory of Southwestern Chinese Medicine Resources, Chengdu University of Traditional Chinese Medicine, Chengdu, Sichuan, China; ^2^ State Key Laboratory of Oncology in South China, Guangdong Provincial Clinical Research Center for Cancer, Sun Yat-sen University Cancer Center, Guangzhou, China; ^3^ Department of Pharmacology, Key Laboratory of Drug-Targeting and Drug Delivery System of the Education Ministry, Sichuan Engineering Laboratory for Plant-Sourced Drug and Sichuan Research Center for Drug Precision Industrial Technology, West China School of Pharmacy, Sichuan University, Chengdu, China

**Keywords:** leonurine, pharmacokinetics, pharmacology, toxicology, clinical trial

## Abstract

Leonurine is an alkaloid unique to the *Leonurus* genus, which has many biological activities, such as uterine contraction, anti-inflammation, anti-oxidation, regulation of cell apoptosis, anti-tumor, angiogenesis, anti-platelet aggregation, and inhibition of vasoconstriction. This paper summarizes the extraction methods, synthetic pathways, biosynthetic mechanisms, pharmacokinetic properties, pharmacological effects in various diseases, toxicology, and clinical trials of leonurine. To facilitate a successful transition into clinical application, intensified efforts are required in several key areas: structural modifications of leonurine to optimize its properties, comprehensive pharmacokinetic assessments to understand its behavior within the body, thorough mechanistic studies to elucidate how it works at the molecular level, rigorous safety evaluations and toxicological investigations to ensure patient wellbeing, and meticulously conducted clinical trials to validate its efficacy and safety profile.

## 1 Introduction

**FIGURE 1:**
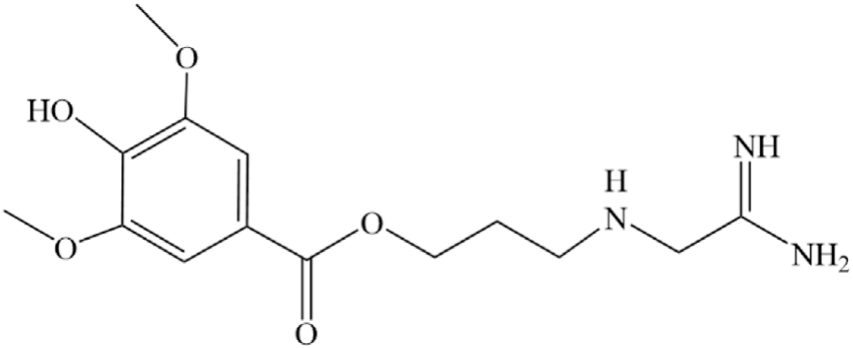
Structure of LEO drawn by ChemDraw.

*Leonurus*, a genus of *Leonurus japonicus* in the Lamiaceae family, is a yearly or biennial botanical drug. The drug is extensively utilized for the treatment of menstrual disorders, dysmenorrhea, amenorrhea, blood stasis, postpartum hemorrhage, postpartum abdominal pain, edema, urination issues, bloody urine, traumatic injury, and skin conditions such as sore carbuncles and swollen poison. It is also recorded in the seventh edition of the European Pharmacopoeia (Y. Z. Zhu et al., 2018). More than 280 secondary metabolites have been isolated from *Leonurus*, including alkaloids, terpenes, flavonoids, phenylpropanoids, polysaccharides, and volatile oils (Miao et al., 2019). Fresh *Leonurus* contains approximately 0.02%–0.12% leonurine (LEO) (Figure 1) (Li et al., 2022), and it is the unique alkaloid of the *L. japonicus* genus as well as the main active metabolite (Liu et al., 2012). Pharmacological studies have uncovered that LEO possesses activities such as uterine contraction, anti-oxidative stress, anti-inflammation, apoptosis regulation, anti-tumor effects, and angiogenesis, and it exhibits a protective effect on the uterus, cardio- and cerebrovascular systems, nervous system, liver, kidneys, skin, bone tissue, and tumors. In recent years, research on LEO has surged, mainly concentrated on cardio- and cerebrovascular protection and neural preservation (Li et al., 2020). This review discusses the extraction methods, synthetic pathways, biosynthetic mechanisms, pharmacokinetic properties, pharmacological effects in various diseases, toxicology, and clinical trials of LEO. We studied publications collected from PubMed, the Web of Science, and the China National Knowledge Infrastructure databases over the past 5 years (2018–2023). There were also a small number of other publications prior to 2018 that provided both insight and critical interpretation.

## 2 Extraction and synthesis of LEO

### 2.1 Extraction and purification from *Leonurus*

LEO was initially isolated from the plant *Leonurus* in 1930, marking its discovery in the realm of pharmacognosy. Then, in 1976, Yeung CH et al. extracted it from comminuted dried plants or fresh leaves. First, they were extracted using a rotary evaporator in an acidic methanol solution of 1% HCl at a low temperature, further extracted in order of hydrochloric acid, diethyl ether, ethanol, and acid methanol, and then separated and purified using alumina and Sephadex G-25 columns. Finally, 50 mg/kg of LEO dried product was obtained. Meanwhile, they discovered that LEO could apparently contract the isolated uterus (Kong et al., 1976). The next year, they identified the specific structure of LEO by IR, NMR, and MS (Figure1) and verified the uterine contraction effect again (Yeung et al., 1977). In recent years, a variety of extraction techniques, like high-performance liquid chromatography (Kuchta et al., 2012), high-speed countercurrent chromatography (Jiang et al., 2017), and acidic ionic liquid ultrasonic-assisted extraction (Cao et al., 2018), have been used in the extraction and separation of the metabolites of natural drug monomers to obtain pure LEO more efficiently and quickly.

### 2.2 Artificial synthesis

**FIGURE 2:**
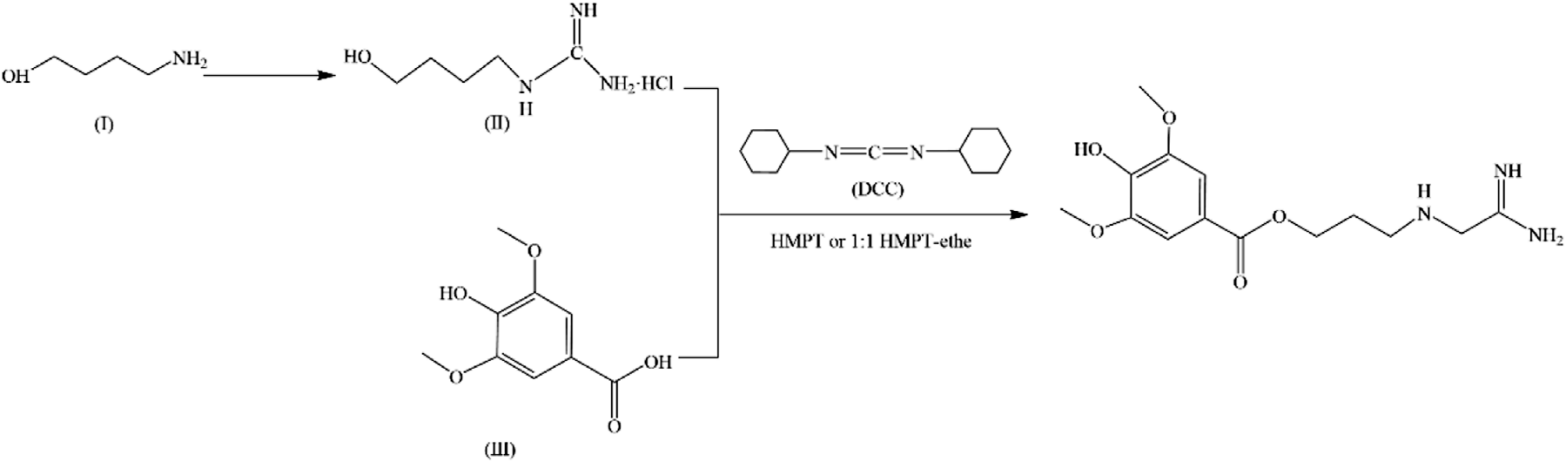
Synthesis of LEO.

**FIGURE 3:**
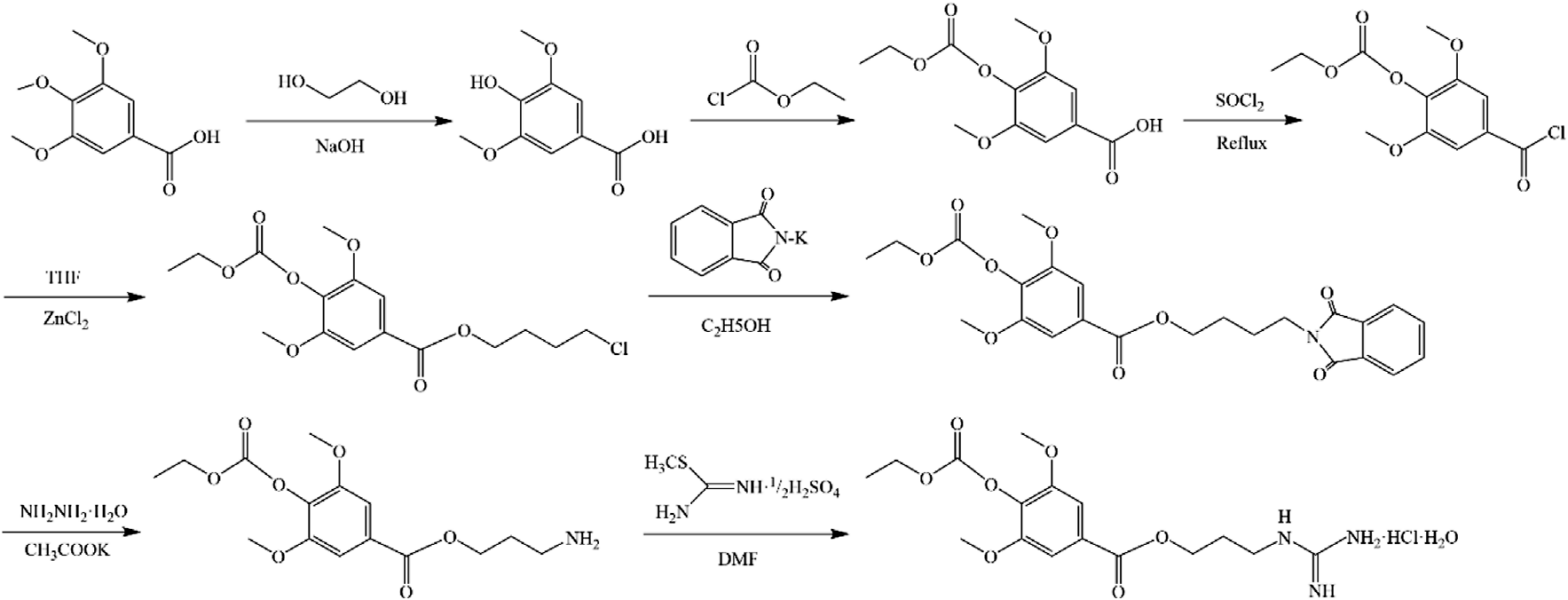
Synthesis of LEO hydrochloride.

In 1979, a simple and high-yield chemical synthesis route was favorably developed that allowed industrial production. In brief, 4-aminobutanol(Ⅰ) was converted to 4-guanidine-1-butanol hydrochloride(Ⅱ), and then the condensation of Ⅱ with syringic acid(Ⅲ) was catalyzed by dicyclohexylcarbodiimide (DCC) in a solution of hexamethylphosphotriamide (HMPT) or a 1:1 HMPT-ether mixture at room temperature for 72 h, resulting in the generation of more than 80% LEO (Figure 2). The product had the same uterine contraction effect as the plant extractions’ in the rat-isolated uterus (Cheng et al., 1979). Wang J et al. synthesized LEO hydrochloride base with 3, 4, 5-trimethoxybenzoic acid as the raw material through seven steps (Figure 3), and the yield reached 11.67%. In addition, they further validated the structure by 1-NMR and MS (Wang et al., 2022).

### 2.3 Biosynthesis

Li et al. conducted multi-omics analysis of the genome and metabolites of *L. japonicus* Houtt. (high content of LEO) and *Leonurus sibiricus* L. (very low content of LEO), revealing the role of *Leonurus* in the biosynthetic pathway and identifying several key rate-limiting enzymes. Arginine in *Leonurus* undergoes several steps, including arginine decarboxylase (ADC) catalysis, amine oxidation, and aldehyde reduction, to produce guanbutol. Meanwhile, syringic acid is activated by UDP-glucosyltransferase (UGT) to form syringoyl glucose. LEO is synthesized by guanbutol and syringoyl glucose together with serine carboxypeptidase-like (SCPL) acyltransferase. Analysis of the subcellular localization of these enzymes suggests that guanbutol and syringoyl glucose may be synthesized in the cytoplasm, whereas the final synthesis step takes place in the vacuole. UGT and SCPL gene amplification and novel functionalization of SCPL jointly determine the specific synthesis and accumulation of LEO in *Leonurus* (Li et al., 2023).

## 3 Pharmacokinetics of LEO

Rats administered LEO at a dose of 5 mg/kg, either through intravenous injection or intragastric administration, showed plasma concentration–time profiles that conform to a two-compartment open pharmacokinetic model. The half-life of LEO was recorded at 1.72 h, with a residence time of 2.17 h, indicating a swift elimination process from the body. Its volume of distribution stood at 24.73 L/kg, suggesting that LEO is primarily distributed in the plasma and extracellular fluids. Upon oral administration of a 50 mg/kg dose of LEO to rats, the drug exhibited rapid absorption, achieving the peak plasma concentration approximately 0.75 h post-administration (Wang et al., 2022). It was mainly distributed in the stomach, liver, kidney, lung, spleen, heart, and pancreas and rarely concentrated in fat and muscle, indicating its limited ability to penetrate the blood–brain barrier. The binding rate of plasma protein (BRPP) in LEO was different between different species, but the BRPP was lower than 80% in general (Zhu 2015). The oral bioavailability was 2.21%, indicating that LEO probably had a high first-pass elimination and enterohepatic circulation (J. Wang et al., 2022). It was determined by the intestinal loop method that LEO had first-pass elimination and the metabolic rate was surpassing 90%. The metabolites of LEO in rat plasma, urine, and bile were sulfate conjugates, aldehyde conjugates, and ester bond hydrolysis products. The main metabolic enzymes involved CYP2D6, CYP1A2, and CYP3A4, participating in Phase I metabolic reactions, and UGT1A1, catalyzing the LEO gluconic acid acidification reaction. Among them, the phase Ⅱ glucuronic acid conjugate, leonurine-10-O-β-D-glucuronide (L-O-G), was the main mode of intestinal metabolism and had the same biological activity as LEO. However, in feces, it was mainly excreted as prototype drugs (Zhu 2015; Zhao et al., 2022). LEO competitively inhibited the activities of CYP1A2 and CYP2D6 (IC_50_ = 18.05 and 15.13 μM) and showed a non-competitive inhibition on the activity of CYP3A4 (IC_50_ = 20.09 μM). Meanwhile, the inhibitory effect of LEO on CYP3A4 was time-dependent. This suggests that there were potential drug interactions between LEO and drugs metabolized by CYP1A2, CYP2D6, and CYP3A4 (Zhao et al., 2021). A detailed overview of the specific pharmacokinetic parameters is provided in Table 1 for further clarification.

**TABLE 1:** Pharmacokinetic studies of LEO.

Route of administration	Species	Dose (mg/kg)	Pharmacokinetic parameter							Reference
			t_1/2z_(h)	T_max_(h)	C_max_(ug/L)	CL_z_(L/h/kg)	V_z_(L/kg)	AUC_(0-t)_ (ug/L*h)	AUC_(0-∞)_ (Ug/L*h)	
i.v	Rats	5	1.74 ± 0.30	2 ± 0	2,359 ± 306.1	10 ± 1.44	24.73 ± 3.58	477.4 ± 25.2	510.9 ± 91.0	J. Wang et al. (2022)
	Rats	15	2.01 ± 1.48	0.08 ± 0	8.50 ± 1.05	3.96 ± 0.29	11.86 ± 9.58	3,410 ± 69	3,800 ± 27	Zhu Q. (2015)
	Dogs	4	1.3 ± 0.4	0.1 ± 0	1,175.6 ± 300.7	4.4 ± 1.2	7.8 ± 1.9	950.1 ± 209.1	954.5 ± 208.9	Zhang J. (2015)
i.g	Rats	50	2.04 ± 12.64	0.75 ± 0.158	43.93 ± 14.89	397.9 ± 0.158	2,141 ± 0.158	105.5 ± 12.3	151.8 ± 98.8	J. Wang et al. (2022)
	Rats	0.29	2.04 ± 0.45	0.99 ± 1.51	1.55 ± 0.52	51.75 ± 7.62	148.6 ± 180.2	5.53 ± 0.77	5.74 ± 0.96	Wen et al. (2019)
	Rats	15	3.09 ± 0.72	0.95 ± 0.37	36.00 ± 23.28	125.54 ± 58.92	519.01 ± 193.18	147.39 ± 77.42	153.94 ± 97.64	Zhu Q. (2015)
	Dogs	20	1.9 ± 0.4	1.0 ± 0.6	115.5 ± 66.0	71.8 ± 39.4	193.7 ± 107.9	352.3 ± 187.3	359.5 ± 191.4	Zhang J. (2015)

## 4 Pharmacodynamics of LEO

### 4.1 Protective effect on obstetrical and gynecological disease

**FIGURE 4:**
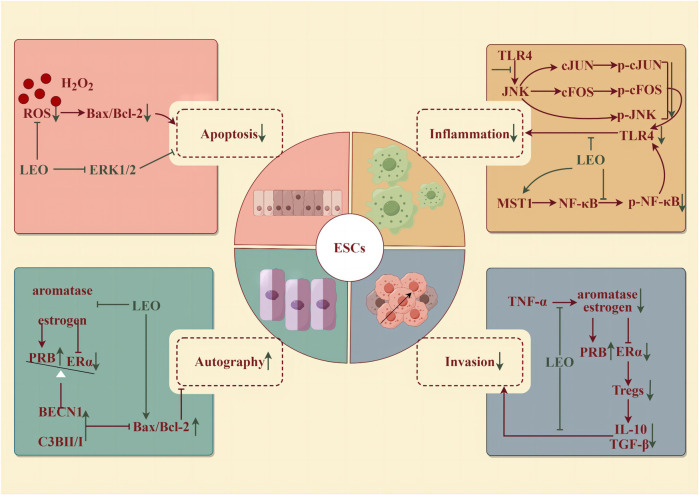
Protective effect of LEO on ESCs (by Figdraw). LEO can reduce the apoptosis and inflammatory response of ESCs, inhibit invasion, and promote the autophagy of ectopic ESCs.

Physiological uterine contractions facilitate the expulsion of menstrual blood during the menstrual period as well as fetal pregnancy or delivery, postpartum hemostasis, and uterine involution. However, an imbalance in the intensity of contractions—whether too forceful or too feeble—can lead to detrimental health consequences. In 1976, it was isolated from *Leonurine* plants and observed to have pharmacological effects on uterine contractions in the rat-isolated uterus (Kong et al., 1976). Only a concentration of 0.4 pg/mL of LEO could trigger regular and significant muscle contractions (Yeung et al., 1977). Then, it was confirmed that the contraction activity corresponded to oxytocin, which could be in a dose-dependent manner to promote the strength of contractions and tension within the uterine muscles and elevate the frequency of contractions (Liu et al., 2018). Simultaneously, LEO harnesses its multifaceted therapeutic potential through its robust antioxidant, anti-inflammatory, and anti-apoptotic characteristics (illustrated in Figure 4).

#### 4.1.1 Anti-endometrial dysfunction

Oxidative stress, a disturbance in the balance between reactive oxygen species (ROS) and antioxidants, within endometrial stromal cells (ESCs)—the fundamental building blocks of the endometrium—has been implicated in the development of several disorders. These include endometritis, endometriosis, eclampsia, and decidualization impairment (Leitao et al., 2010). Specifically, endometritis represents an infectious and inflammatory condition of the endometrium, typically resulting from bacteria through the cervical canal or vaginal opening (Singh and Sethi, 2022). In the case of chronic endometritis, the pathology is described as structural destruction of the endometrium and an uncontrolled inflammatory cascade (Lin et al., 2021). LEO pretreatment for 4 h (25 and 50 μM) could effectively reverse the detrimental effects of H_2_O_2_-induced oxidative injury in human ESCs, including restoring morphological changes, mitigating cellular damage, reducing DNA fragmentation, suppressing apoptosis, and alleviating the accumulation of ROS. Further studies discovered that LEO pretreatment markedly decreased the phosphorylation of ERK1/2 (Li et al., 2019). Meanwhile, LEO effectively alleviated inflammation by reducing levels of IL-1β, IL-6, IL-8, TNF-α, iNOS, and COX2 induced by LPS in both mouse and human ESCs, whether administered as pretreatment or post-treatment. LPS also accelerated JNK, cFOS, cJUN, p65 phosphorylation, and IκB expression in human ESCs, which were dose-dependently controlled by LEO (Lin et al., 2021).

Endometriosis (EMS) is a disorder characterized by the abnormal growth of endometrioid tissue outside the uterus, leading to the formation of scar tissue. This misplaced tissue can provoke chronic pelvic discomfort, manifesting as pain during menstruation, intercourse, and urination, and even lead to infertility (Bulun et al., 2019). The prevailing pathogenic theory is the retrograde menstruation hypothesis, which states that as menstrual blood flows out of the body through the cervix and vagina during menstruation, part of the menstrual blood containing endometrial cells flows backward from the fallopian tubes to the pelvis to implant in the peritoneum and abdominal organs, where it proliferates, causing chronic inflammation and adhesion formation (Vercellini et al., 2014). EMS is an estrogen-dependent disease; that is, excessive estrogen and deficiency of progesterone levels would enhance the survival ability of ectopic endometrium. The accumulation of pro-inflammatory factors such as TNF-α can boost the proliferation of ectopic endometrial stromal cells (eESCs) and thus exacerbate EMS. LEO could inhibit the progression of EMS by inhibiting the aromatase-estrogen receptor (ER) α activated by TNF-α, increasing the expression of progesterone receptor subtype B (PRB), and promoting autophagy and apoptosis of eESCs from the mouse model (7.5 and 15 mg/kg/d for 7 days) and patients (Lin et al., 2022). In addition, the occurrence of EMS is closely related to immune system disorders (Wang et al., 2021). Estrogen-ERα-induced Tregs expansion and the accumulation of cytokines (IL-10 and TGF-β1) produced by Tregs were found in the peritoneal fluid of EMS patients (Xiao et al., 2020). LEO effectively diminished the differentiation and cytokine secretion of Tregs by suppressing estrogen-ERα signaling. After 48 h of pretreatment with 200 μM, LEO notably improved the invasion and survival of eESCs enhanced by Tregs. *In vivo*, LEO administered at both low and high doses (7.5 and 15 mg/kg/d for 7 days) inhibited the growth of EMS model mice abdominal ectopic lesions and lowered the EMS Tregs accumulation (Li et al., 2022b).

Pre-eclampsia (PE) occurs in the third trimester of pregnancy (>20 weeks) and manifests as new-onset hypertension with urinary protein, which can rapidly develop into serious and irreversible complications. Multiple factors contribute to the development of PE, including impaired remodeling of spiral arterioles, hypoxia, oxidative stress, poor decidualization of the uterus, abnormal natural killer (NK) cells at the maternal–fetal interface, genetic and environmental factors leading to early placental abnormalities, imbalances in circulating angiogenic factors in mid-to-late pregnancy, and changes in inflammatory cytokines and immune cells (Rana et al., 2019; Chappell et al., 2021). Among them, abnormal trophoblast invasion is a vital element in the pathogenesis of PE. Abnormalities in the trophoblast itself may result in inadequate transformation of the superficial placenta and spiral arteries, ensuring placental ischemia and maternal PE. LEO diluted LPS-induced high levels of inflammatory cytokine TNF-α and the expression of p-p65 in the human placental trophoblast HTR8/Svneo cells with increasing concentrations (0, 5, 10, and 20 μM for 6 h). In addition, LEO significantly upregulated the level of MST1, the negative regulator of NF-κB signaling, in LPS-induced trophoblast HTR8/Svneo cells. In contrast, in MST1−/−cells, LEO had little anti-inflammatory effect. Altogether, LEO may inhibit trophoblast cell inflammation by upregulating the level of MST1, which provides important research and clinical implications for trophoblast inflammation-induced PE (Zong and Zhao, 2021).

#### 4.1.2 Anti-premature ovarian insufficiency

Premature ovarian insufficiency (POI) refers to the depletion of ovarian follicles, reproductive ability, and endocrine dysfunction or loss in women before the age of 40. As a result of low estrogen and high gonadotropins, disorders such as irregular menstruation, pregnancy failure, and menopausal syndrome occur (Chon et al., 2021). The specific pathogenesis of POI is still unclear, but inflammatory aging (characterized by a chronic, gradually increased pro-inflammatory state with age) has been confirmed to play an important role during the attack of POI. The expressions of NLRP3 inflammasome, caspase-1, and IL-1β were increased in POI patients (Navarro-Pando et al., 2021), and the pro-inflammatory factor in the POI model mice’s serum level was higher than that in normal mice (Huang et al., 2019). LEO hydrochloride administration by intraperitoneal injection (7.5, 15, and 30 mg/kg/d for 28 days) could protect ovarian function and maintain serum hormone levels in POI mice induced by cyclophosphamide (150 mg/kg/week). In the range of doses tested, the dose of 30 mg/kg was optimal for the regulation of serum hormones and protection of reproductive organ weight and follicle number. However, the low- and medium-dose (7.5 and 15 mg/kg) groups had a stronger effect on improving the fertility of POI mice, significantly increasing the number of live fetuses and implantation. LEO hydrochloride could inhibit the activation of NLRP3 inflammasome, caspase-1, and GSDMD in ovarian tissues and granulosa cells, reduce serum IL-18 and IL-1β levels, and thus continuously resist cyclophosphamide-induced ovarian injury (Chi et al., 2023).

### 4.2 Protective effect on cardio-cerebrovascular diseases

**FIGURE 5:**
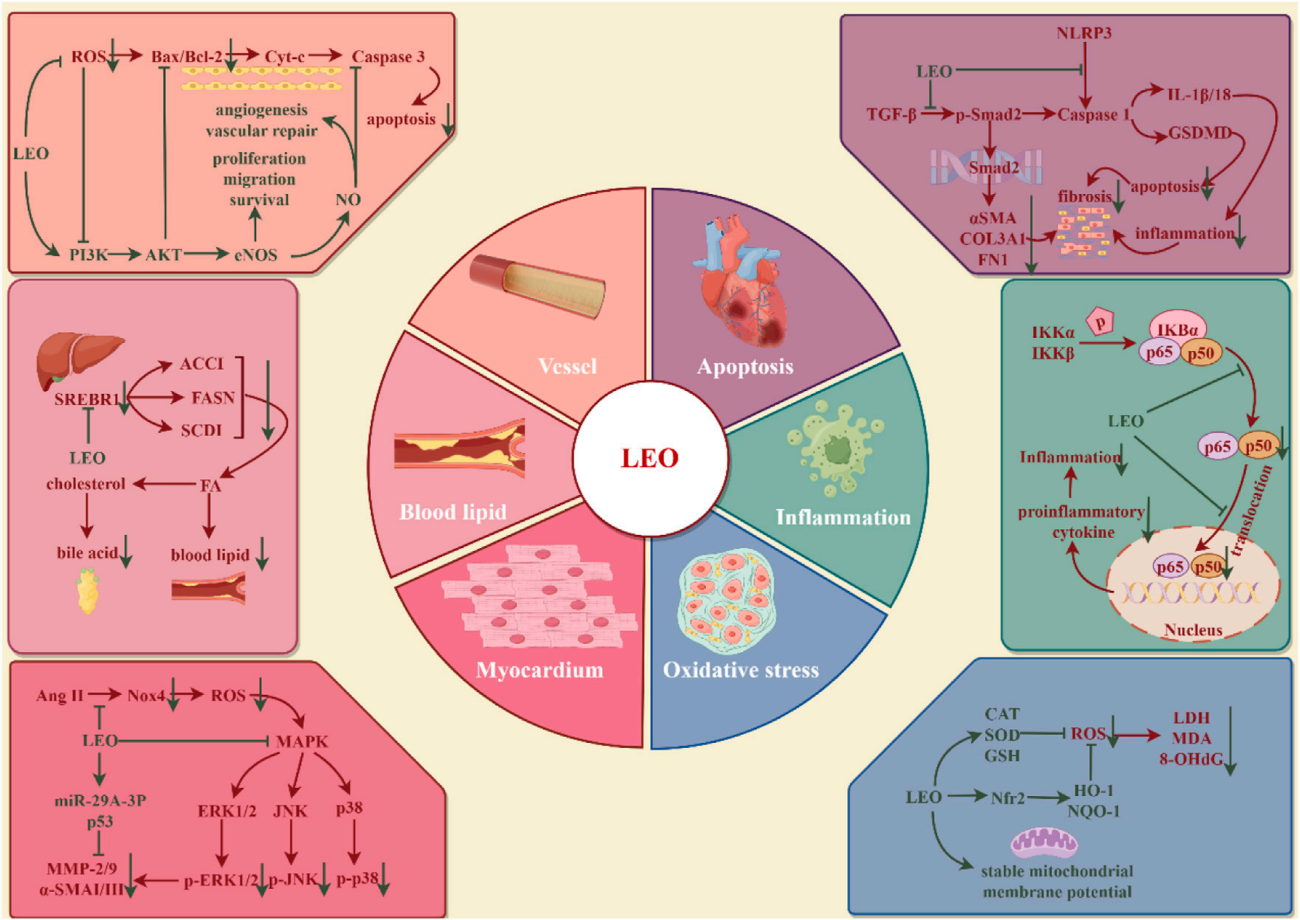
Protective effect of LEO on cardio-cerebrovascular system (by Figdraw). LEO can inhibit cardio-cerebrovascular inflammation, oxidative stress, reduce cardiomyocyte apoptosis and myocardial fibrosis, promote vascular endothelial homeostasis, and restore dyslipidemia.

Cardio-cerebrovascular diseases (CCVDs) encompass both cardiovascular and cerebrovascular disorders, characterized by high morbidity and mortality, posing a significant health threat globally (Su et al., 2021). Cardiovascular disease (CVD) is currently the world’s leading cause of death, with more deaths occurring each year from CVD than from any other cause of death (Santo and Redfern, 2020). In addition to the therapeutic drugs recommended by the WHO, such as aspirin, beta-blockers, angiotensin-converting enzyme inhibitors, statins, and surgical treatment, traditional Chinese materia medica has gained widespread acceptance in various countries for the prevention and management of CVD (Hao et al., 2017). Extensive research has validated LEO’s protective role in CCVDs (illustrated in Figure 5). LEO is now advancing into clinical trials as a novel therapeutic approach for CCVDs.

#### 4.2.1 Anti-atherosclerosis

Atherosclerosis (AS) is a chronic lipid-driven arterial wall inflammatory disease that damages the coronary, cerebral, or internal carotid arteries, leading to acute ischemia, vascular thrombosis, and tissue infarction (Badimon and Vilahur, 2014). Endothelial cells are monolayer surface cells that line the inner surface of blood vessels and participate in the processes of blood vessel formation, blood coagulation, blood vessel contraction, blood vessel dilatation, and inflammation (Xu et al., 2021). Vascular lesions, including endothelial dysfunction, massive lipid deposition in the intima, increased innate and adaptive immune responses, proliferation of vascular smooth muscle cells, and remodeling of the extracellular matrix, contribute to the formation of atherosclerotic plaques (Badimon and Vilahur, 2014). Endothelial dysfunction runs through the whole process of early plaque formation, plaque progression, and rupture of unstable plaques (Xu et al., 2021). Human umbilical vein endothelial cells (HUVECs) were exposed to H_2_O_2_ (200 μM) to induce oxidative stress injury and angiogenesis disorders. After treatment with LEO (2.5, 5, and 10 μM) for 24 h, cell proliferation, migration, and tube formation were promoted. LEO significantly downregulated oxidative stress factors, such as ROS, MDA, and LDH, and upregulated SOD, the activity of intracellular antioxidant factors, and NO, the vascular endothelial regulation factor. Meanwhile, LEO treatment significantly promoted the phosphorylation levels of PI3K, Akt, and eNOS and the expression level of Bcl2 but decreased the expression levels of Bax and caspase-3 (Liao et al., 2021). Lowering LDL-c and TG and increasing HDL-c are the primary therapies for controlling atherosclerotic disease (Pérez de Isla et al., 2017). The improvement effect of LEO on blood lipids is similar to that of positive medicine statins and has a higher security. ApoE^−/−^ mice, New Zealand white rabbits, and aged Rhesus monkeys with high-cholesterol diets were used to build the AS models. The results showed that 20 mg/kg LEO could reduce serum TC, TG, and LDL levels in mice but did not affect HDL, 16 mg/kg LEO reduced serum TC and TG levels in rabbits, and 8 mg/kg LEO plunged TG levels but was invalid in LDL. In rhesus monkeys, which are more closely related to humans, 10 mg/kg LEO showed varying degrees of reduction in serum TC and LDL levels. Additionally, the expression of fatty acid genes, such as fatty acid synthase and stearoyl-CoA desaturase-1, was also inhibited (Suguro et al., 2018). Therefore, LEO may be an alternative drug for patients with hypercholesterolemia who are intolerant to statins. Atherosclerotic plaque rupture, which leads to high-risk coronary thrombosis, is primarily characterized by a thin fibrous cap, a lipid-rich necrotic core, and a high inflammatory cell count. However, LEO (20 and 40 mg/kg/d for 91 days) was able to increase fibrous cap thickness and collagen content to enhance plaque stability and prevent stroke and myocardial infarction, which is mediated by regulating the balance of the NOS-NO system to maintain vascular homeostasis and inhibiting inflammatory response through NF-κB in ApoE−/−mice (Ning et al., 2020).

#### 4.2.2 Anti-myocardial infarction and anti-myocardial ischemia-reperfusion injury

Myocardial ischemia (MI) is a symptom of myocardial necrosis caused by persistent or acute ischemia of the coronary artery, which mostly occurs based on coronary atherosclerotic stenosis. The rupture of atherosclerotic plaques, accompanied by thrombosis, causes continuous hypoxia of myocardial cells, resulting in myocardial cell necrosis (Lu et al., 2015). At present, MI is mostly treated through increasing coronary blood flow and limiting or reducing infarct size, like the use of thrombolytic drugs, primary percutaneous coronary intervention (PPCI), and surgical bypass surgery. Studies have exhibited that LEO inhibits intracellular Ca^2+^ overload and apoptosis in hypoxic cells, which may be the reason for its cardioprotective effect (Liu et al., 2009). The AMI model was established by surgical ligation of the left anterior descending coronary artery in rats. Intervention with LEO at 15 mg/kg for 7 days before surgery, followed by administration on the day of surgery, resulted in a decline in myocardial infarct size and significant reductions in CK-MB activity and Tn-I concentrations. The study also integrated metabolomics and network pharmacology to analyze the mechanism of LEO against ischemic heart disease, especially AMI. Thirty-two differential metabolites were detected in the plasma of AMI rats, and 16 core genes were measured by network pharmacology. Meanwhile, the compound-reaction-enzyme-gene network was constructed. Six key targets (GSR, CYP2C9, BCHE, GSTP1, TGM2, and PLA2G2A) were identified in the network map, corresponding to seven differential metabolites (glycerophosphatidylcholine, lysophosphatidylcholine, phosphocholine, linoleic acid, 13HPODE, tryptophan, and glutamate) and involving four important metabolic pathways (glycerol phosphate metabolism, linoleic acid metabolism, tryptophan metabolism, and glutamate metabolism). Glycerol phosphate metabolism and tryptophan metabolism pathways have been demonstrated in AMI clinical trials, which harmonize with the results of metabolomics studies (Rong et al., 2022). Moreover, it is found that the damage to cardiomyocytes due to the restoration of ischemic myocardial blood flow (myocardial ischemia-reperfusion injury, MIRI) exceeds the injury of ischemia and hypoxia themselves. In the process of myocardial ischemia-reperfusion, excessive oxidative stress, intracellular Ca^2+^ overload, physiological PH changes, inflammation, and other mechanisms jointly result in myocardial cell death in MIRI (Hausenloy and Yellon, 2013). LEO preconditioning at doses of 7.5 and 15 mg/kg could inhibit the release of infarct-related enzymes such as serum creatine phosphokinase, aspartate aminotransferase, and lactate dehydrogenase, prominently reduce the infarct size, maintain the morphology of myocardial cells, and restore heart function in I/R mice. Neonatal rat ventricular myocytes (NRCMs) were subjected to hypoxia/reoxygenation (H/R) every 12 h in an incubator to create an I/R model *in vitro*, and LEO intervention (0.1, 1, and 10 μM) was performed before modeling. The results suggested LEO reduced intracellular ROS levels, apoptosis, and phosphorylation of Ak, p38, and JNK while enhancing the viability of NRCMs (Lu et al., 2023).

#### 4.2.3 Anti-cardiac fibrosis

Myocardial fibrosis (CF) is a common pathological change in many myocardial diseases and runs through the whole process of heart injury. CF refers to a pathological change in which extracellular matrix (ECM) proteins are deposited and cardiac interstitial dilated (Frangogiannis, 2021), which leads to the accumulation of scar tissue, a decrease in myocardial elasticity, and the influence of cardiac output, eventually arousing cardiac insufficiency. Increased production or decreased degradation of the ECM, for example, collagen, a-smooth muscle actin (a-SMA), fibronectin, elastin, and fibrillin, all contribute to the development of fibrosis. This may be related to the abnormal expression of matrix metalloproteinases (MMP) that degrade the ECM, its inhibitors, tissue inhibitors of metalloproteinases (TIMPs), and pro-fibrotic mediators such as transforming growth factor-β (TGF-β) and platelet-derived growth factor (PDGF) (He et al., 2013). Pretreatment with LEO (10 and 20 μM) for 4 h reduced Ang-Ⅱ-induced NADPH oxidase 4 (Nox4) activation, ROS production, MMP 2/9, and a-SMA Ⅰ as well as Ⅲ collagen expression in the neonatal rat cardiac fibroblasts. Meanwhile, LEO (7.5, 15, and 30 mg/kg/d for 42 days) could inhibit myocardial fibrosis and decrease Nox4 expression, ROS and NF-κB activation, and plasma MMP-2 activity in post-MI rats (Liu et al., 2013, 4). The MiR-29 family has an anti-fibrotic function and is involved in the inhibition of ECM synthesis (He et al., 2013). Another study exhibited that all mature miR-29 family members were downregulated in the myocardial tissue of post-MI mice induced by isoprenaline, while LEO treatment (25, 50, and 100 mg/kg/d for 48 days) not only inhibited fibroblast activation and collagen synthesis but also noticeably upregulated the expressions of miR-29a-3p and p53. However, the knockdown of miR-29a-3p or PFT-α (a p53 inhibitor) completely abolished the therapeutic effect of LEO on myocardial fibrosis, as evidenced by the upregulation of TGF-β, collagen type III, and collagen type I protein levels in CFs (Li et al., 2022; Xi et al., 2023). Furthermore, a novel mechanism of LEO against myocardial fibrosis was discovered. Pretreatment with 20 μM LEO for 4 h could repress the phosphorylation of Smad2 in H9c2 cells stimulated with 20 ng/mL TGF-β for 30 min or 48 h, indicating its potential role in the regulation of the TGF-β/Smad2 signaling pathway. At the same time, LEO affected the progression of CVD, as indicated by a reduction in fibrosis area and collagen expression, by interfering with pyroptosis-related molecules, like the lowered expression of caspase-1, cleaved caspase-1, GSDME, and cleaved GSDME *in vivo* and *in vitro* (Li et al., 2022b; Li et al., 2022c). To sum up, LEO affects the development of CF in multiple dimensions.

#### 4.2.4 Anti-ischemic stroke

Ischemic stroke (IS) is a disease that occurs when the internal carotid and vertebral arteries are narrowed and blocked, promoting obstruction of blood supply to the brain and causing damage to brain tissue. The pathophysiological mechanisms of IS cover cellular excitotoxicity, oxidative stress, cell death processes, and neuroinflammation, as well as various intricate signaling pathways (Qin et al., 2022). Rats were exposed to photochemicals (OGD) for 2 h to induce the IS model. ROS and MDA levels increased, and SOD activity, CAT, and GSH content decreased. However, pretreatment with LEO (50, 100, and 200 μg/mL) offset these transformations in a dose-dependent manner (Deng et al., 2022). Simultaneously, LEO increased the expression of vascular endothelial growth factor (VEGF) in neurons, astrocytes, and endothelial cells in IS mice that were administered LEO at a dosage of 10 mg/kg for 2 h after pMCAO via intraperitoneal injection. The LEO upregulated nuclear Nrf-2 protein and increased total Nrf-2 mRNA level and protein expression but had no therapeutic effect on Nrf-2−/−mice (Xie et al., 2019). Furthermore, LEO not only inhibited the increase in NO content and the expression of NOS/iNOS/cNOS activated by OGD in PC12 cells or IS rats’ serum and brain tissues (Deng et al., 2022) but also increased the Bcl-2/Bax ratio to inhibit neuronal apoptosis (Zhang et al., 2019).

### 4.3 Neuroprotective effects

**FIGURE 6:**
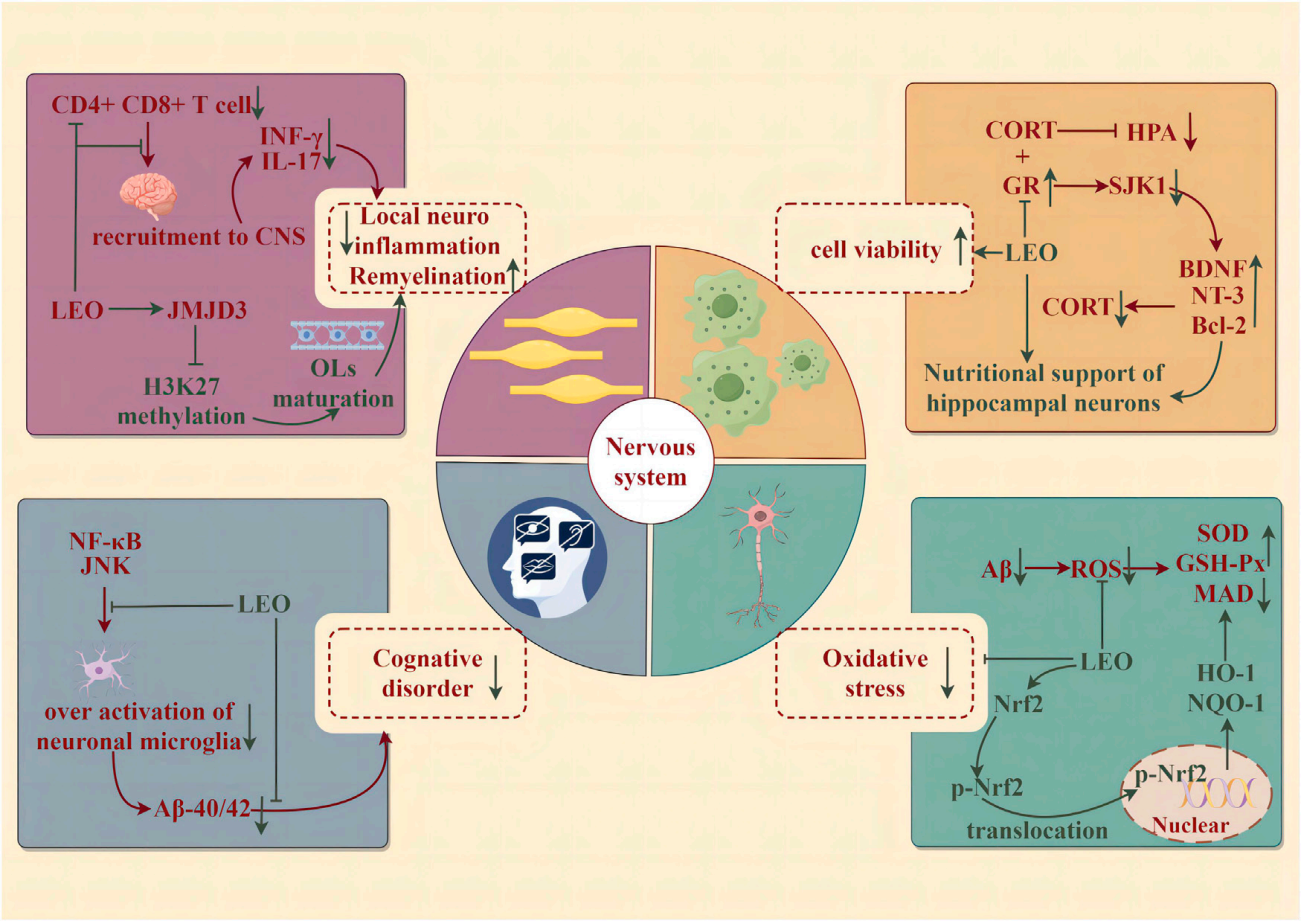
Protective effect of LEO on the nervous system (by Figdraw). LEO significantly reduces neuroinflammation and oxidative stress in lesions, improves nerve cell viability, and effectively improves cognitive impairment caused by the disease.

Neurodegenerative diseases (NDDs) are a group of neurological diseases with progressive loss of neurons in the central nervous system (CNS) and peripheral nervous system (PNS) cells. NDDs are associated with aging and may be detected along with age: Alzheimer’s disease (AD) and Parkinson’s disease (PD), or due to genetic mutations affecting CNS cellular function: Huntington’s disease, early-onset AD or PD, and amyotrophic lateral sclerosis. The potential of LEO to improve the syndrome and alleviate the progression of NDDs has attracted the attention of researchers and the medical community (illustrated in Figure 6).

#### 4.3.1 Anti-Alzheimer’s disease

Alzheimer’s disease is characterized by an insidious onset accompanied by progressive development. Clinical features include cognitive impairment in learning, memory, language, visuospatial skills, executive dysfunction, and personality and behavioral changes. The β amyloid protein (Aβ) cascade hypothesis is widely accepted: it has been recognized as the initiating factor of neuronal lesions that the deposition of generalized brain atrophies amyloid plaques composed of Aβ and neuronal fibrillary tangles (NFTs) composed of hyperphosphorylated microtubule-associated protein tau in the cortex and limbic regions of the brain (Khan et al., 2020). In summary, changes in neuronal cell metabolism because of oxidative stress, neuroinflammation, neurotransmitter imbalance, and gene mutations lead to the continuous accumulation of Aβ and NFTs in the brain, and then neuronal cell death eventually causes the continuous progression of NDDs (Maltsev et al., 2011). The results of the novel object recognition test and water maze test indicated that LEO (150 mg/kg/d for 60 days) could significantly improve the cognitive dysfunction of APP/PS1 mice. The evident nuclear rupture and less intact Nissl material in the hippocampal CA1 region of APP/PS1 mice were improved significantly. Likewise, LEO remarkably lessened the contents of Aβ1-40 and Aβ1-42. In addition, LEO exerted its anti-oxidative ability by augmenting the expression of nuclear-Nrf-2, cytoplasmic-Nrf-2, HO-1, and NQO1 at the gene and protein levels in APP/PS1 mice, which was characterized by the downregulation of ROS and MDA and the upregulation of SOD and GSH-Px (Xie et al., 2023).

#### 4.3.2 Anti-depression

Depression is a common mental disorder, which is described as a downcast mood, delayed thinking or action, and poor cognition, accompanied by somatic symptoms such as sleep disorders. The hypothalamic–pituitary–adrenal (HPA) axis is a self-regulated stress response system that plays an important role in central stress, inflammatory response, and neurological function (Cb and Ww, 2005). HPA axis hyperfunction is thought to be associated with impaired feedback inhibition mediated by endogenous glucocorticoids, which is relative to the combination of glucocorticoid receptors (GRs) and mineralocorticoid receptors (MRs). Circulating glucocorticoids bind to GRs both within and outside the brain, and activated GR, in turn, induces feedback inhibitory signals, thus leading to the reduction of HPA axis activity (Pariante and Lightman, 2008). Corticosterone (CORT) can restrain the activity of PC12 cells to establish a depression model. However, LEO abolished the inhibitory effect of CORT on cells and stimulated cell viability in a concentration-dependent manner (10, 20, 40, 60, 80, and 100 μM, 24 h), with the maximal pro-survival effect at 60 μM. At the same time, total neurite growth, maximum neurite length, and cell area, which represented an improvement in the morphological characteristics of neuronal development, were increased in PC12 cells after LEO administration. Following the administration of the SGK1 inhibitor, GSK650394, this promoting impact on neurons was notably augmented. Conversely, when the GR inhibitor, RU486, was applied, it diminished the efficacy of LEO on neurons. Further studies uncovered that LEO could increase GR content in PC12 cells after CORT treatment, thereby enhancing the negative feedback inhibition of the HPA axis, upregulating the levels of neurotrophic factors such as BDNF and NT-3 to heighten neurotrophic support for hippocampal neurons, and reducing the expression of downstream targets of GR such as serum/glucocorticoid-regulated kinase 1 (SGK1), thereby dwindling neuronal morphological changes induced by derived CORT in the brain (Meng et al., 2019).

#### 4.3.3 Anti-multiple sclerosis

Multiple sclerosis (MS) is a chronic autoimmune disease. The typical pathological feature of this disease is primary oligodendrocyte (OL) damage, demyelination, and axonal damage triggered by T cell-mediated inflammation associated with progressive neurodegeneration (Liu et al., 2019). In the CNS, deriving from oligodendrocyte precursor cells (OPCs), OL dynamically and continuously generates myelin. When brain injury occurs, OPCs are activated, recruited, and differentiated to regenerate myelin sheaths, thereby repairing the damage (Liu et al., 2019). The encephalitis-causing T cells are first stimulated in the periphery, then migrate and infiltrate into the CNS, and are reactivated by antigen-presenting cells to produce inflammatory cytokines. Experimental autoimmune encephalomyelitis was induced by the myelin peptide MOG35-55 (EAE) to mimic the autoimmune disease of human MS (Constantinescu et al., 2011). LEO (60 mg/kg) treatment of EAE mice significantly reduced the content of CD4^+^ and CD8^+^ T cells in the CNS and the corresponding CD4^+^ T-cell infiltration producing IFN-γ and IL-17. Nevertheless, it did not affect the activation of peripheral T lymphocytes or the activation of microglia/macrophages. LEO appears to act directly on CNS resident cells to suppress local neuroinflammation and, in turn, the infiltration of encephalitic T cells into the CNS. Mechanistically, LEO may promote the maturation and differentiation of OPCs into OLS by upregulating the expression of histone 3 methylase JMJD3, which is involved in OL differentiation, thereby accelerating the regeneration of injured myelin in EAE mice (Jin et al., 2019).

### 4.4 Anti-tumor effects

Cancer persists as a formidable challenge, characterized by its alarmingly high morbidity and mortality rates and low survival prospects. Many metabolites in natural drugs have been found to possess high anti-cancer activity and low side effects. They act on many forms of tumor-regulated cell death, including apoptosis, necroptosis, autophagy, ferroptosis, pyroptosis, necrosis, parathyroid hormone, entosis, lysosome-dependent cell death, NETosis, oxiptosis, alkaliptosis, cuproptosis, and disulfidptosis (Han et al., 2024). LEO has a broad-spectrum anti-tumor capacity, which can inhibit the proliferation, invasion, and migration and induce apoptosis and autophagy of various tumor cells *in vitro* and *in vivo* (illustrated in Table 2) (Cao et al., 2023).

**TABLE 2:** Effects of LEO on tumors.

Cancer	Model		Effect		Method	Reference
	Vitro	Vivo	Vitro	Vivo		
CML	Human CML cell lines K562 and KU812	Nude mice	(−) proliferation, migration, and invasion (+) apoptosis	(−) tumor growth	(+) SOCS5 (−) miR-18a-5p	H.-M. Liu et al. (2021)
AML	Human AML cell lines HL-60 and U-937	Nude mice	(−) proliferation (+) apoptosis	(−) tumor growth	(−) PI3/Akt	Zhuang et al. (2021)
ALL	Human ALL cell lines NALM6, Molt, and 697	NRG mice	(−) proliferation (+) apoptosis	(−) tumor growth	(−) PI3/Akt	Schramm et al. (2023)
PCa	Human PCa cell lines PC-3 and DU145	Nude mice	(−) proliferation (+) apoptosis	(−) tumor growth	Regulates the cell cycle and induces cell cycle arrest; (+) LC40A1; (−) miR-18a-5p	B. Liang, Cui, and Zou, (2022)
Breast cancer	Human breast cancer cell lines MDA-MB-231 and SK-BR-3	---	(−) proliferation, migration, invasion, and angiogenesis (+) apoptosis	---	(−) PI3/Akt/mTOR	J. Tian, Peng, and Wang (2023)
Cervical cancer	Human cervical cancer cell lines C33A and MS751	---	(−) proliferation (+) apoptosis	---	Induces cell cycle arrest	Lin et al. (2020a)
MM	Mo DCs from HDs and MM patients	---	(+) maturation and activity of mo CDs	---	(+) expression of CD40 and CD83; regulates the arachidonic acid pathway	Chen et al. (2023b)

#### 4.4.1 Anti-leukemia

Leukemia, also known as “blood cancer,” is a type of malignant tumor disease of hematopoietic stem cells (Whiteley et al., 2021). The hallmark cytogenetic feature of chronic myeloid leukemia (CML) is the Philadelphia chromosome t (9; 22) (q34; q11.2), which results in a BCR-ABL1 chimeric gene. This fusion enables multiple mitotic signaling pathways, leading to the expression of the BCR-ABL cancer protein and the activation of the tyrosine kinase (Cortes et al., 2021). Tyrosine kinase inhibitors can significantly improve the clinical symptoms in patients with CML, but numerous patients become resistant and intolerant to them (Hochhaus et al., 2020). Studies have proved that LEO restrained the proliferation, migration, and invasion of CML cells *in vitro* and promoted endogenous apoptosis of cancer cells in a time–dose-dependent manner (0, 0.05, 0.1, 0.2, 0.4, 0.8, 1.4, and 2.0 mM for 24 h or for 12, 24, 36, 48, and 60 h at 0.4 mM). Following a 24-h treatment, the IC_50_ values for LEO in inhibiting cell viability were found to be 0.773 mM in K562 cells and 0.882 mM in KU812 cells. Furthermore, LEO could enhance the sensitivity of imatinib (a tyrosine kinase inhibitor) to CML cells, and combination therapy could significantly inhibit the activity of CML cells. LEO (150 mg/kg/d for 28 days) significantly inhibited the growth of CML transplanted tumors in mice, and the volume and weight were both reduced by LEO. We also found high miR-18a-5p and low SOCS5 expression in cell and tumor tissues, which were extremely reversed by LEO (Liu et al., 2021).

Acute myeloid leukemia (AML) is typified by the accumulation of immature myeloid precursor cells (myeloblasts) in the bone marrow and peripheral blood; on the contrary, the differentiation declines on red blood cells, platelets, and leukocytes (Khwaja et al., 2016). The results displayed that LEO extremely controlled the proliferation of AML cell lines HL-60 and U-937 in a time–dose-dependent manner (1, 2, 5, 10, 20, 50, and 100 μM for 24 h or 48 h). The IC_50_ values for viability inhibition of HL-60 cells were recorded as 28.6 μM (24 h) and 11.3 μM (48 h). For U-937 cells, the IC_50_ values were observed at 17.5 μM (24 h) and 9.0 μM (48 h). The IC_50_ values of LEO in suppressing the viability of HL-60 and U-937 cells were notably higher than those of methotrexate (MTX) and vincristine. The growth of transplanted tumors in animals was also curbed (15, 30, and 60 mg/kg/d for 20 days). Similarly, LEO in combination with paclitaxel or vincristine simultaneously suppressed the activity of HL-60 and U-937 cells. LEO stimulated both endogenous and exogenous apoptotic programs at concentrations of 2, 5, and 10 μM, enhanced the Bax/Bcl-2 ratio and the expression of Cyt C, activated caspase-1, caspase-8, and caspase-9 in the cytoplasm, and finally induced the apoptosis of AML cells. Moreover, LEO decreased the phosphorylation of PI3K and Akt to resist cell proliferation in HL-60 and U-937 (Zhuang et al., 2021).

Likewise, LEO is also a potential medicine for acute lymphoblastic leukemia (ALL). LEO could constrict the activity of several different ALL cell lines (NALM6, MOLT4, and 697 cells) with IC_50_ values in the range of 1.2–4.4 uM and diminish the level of NALM6 leukemia cells in NRG mice (Schramm et al., 2023).

#### 4.4.2 Anti-prostate cancer

Prostate cancer (PCa) is one of the most common malignant tumors in men. There are marked geographic and ethnic differences in PCa morbidity, with the highest rates in North America, Western and Northern Europe, and Australia and a greater risk of early PCa in men of African or Caribbean descent. PCa risk increases strongly with age (Liang et al., 2022; Rebbeck, 2017). LEO can regulate the distribution of tumor cell cycles, induce cell cycle arrest, and effectively block cancer cell proliferation, migration, and invasion. Different concentrations of LEO (200, 400, and 800 μM) prompted cell cycle arrest in the G1 phase in PC3 and DU145 cells and inhibited the expression of CDK2 and cyclin E, the major regulators that promote the G1 to S phase transition. The most obvious effect was observed at a concentration of 800 μM in the tested range. LEO caused cell apoptosis by increasing the ratio of Bax/Bcl-2. Solute carrier family 40 member 1 (SLC40A1), also known as the iron transport protein (a tumor-suppressor protein), is closely related to the process of generality cancers (Liang and Ferrara, 2020). MiR-18a-5p manages the proliferation and apoptosis of tumor cells by regulating the expression of its downstream target gene ferroportin (Wu et al., 2017). After the PC3 and DU145 cells were treated with LEO, the expression of miR-18a-5p was lower, while that of ferroportin was higher. As verified by siRNA knockdown of ferroportin, LEO restrained the growth of PCa cells dependent on upregulating ferroportin. Simultaneously, LEO (150 mg/kg, twice a week, for 5 weeks) narrowed the weight and volume of transplanted tumors in mice. The corresponding changes in miR-18a-5p and ferroportin levels were consistent with the results *in vitro* (Liang et al., 2022).

#### 4.4.3 Anti-breast cancer and anti-cervical cancer

Breast cancer and cervical cancer are the third- and fourth-largest malignant diseases that seriously threaten women’s health. Breast cancer and cervical cancer are prone to recurrence and metastasis and seriously affect the physical and mental health of patients (Buskwofie et al., 2020; White et al., 2021). The results of the CCK8 assay, colony formation assay, and colony formation assay showed that LEO significantly inhibited MDA-MB-231 or SK-BR-3 cell proliferation at 400–800 μM concentrations. Moreover, in this dose range, LEO dose-dependently inhibited cell migration, invasion, and angiogenesis (Tian et al., 2023). High-risk human papillomavirus persistent infection and genetic factors are the principal causes of cervical cancer (Revathidevi et al., 2021). Cisplatin-based neoadjuvant chemotherapy and concurrent chemoradiotherapy have been widely used to treat advanced or recurrent cervical cancer. However, some patients are resistant to cisplatin and are prone to recurrence and poor prognosis. LEO suppressed the growth of cervical cancer cell lines C33A and MS751 in a time- and dose-dependent manner (0, 200, 400, 800, 1,200, 1,600, and 2000 μM for 24 h and 48 h). In addition, LEO could enhance the inhibitory effect on cell proliferation of cisplatin and 5 μM cisplatin plus 800 μM LEO had the synergistic anti-proliferative function (CI = 0.67), which arrested the cell cycle in the G1 phase, induced the expression of cleaved caspase-1 and poly ADP-ribose polymerase protein, and elevated cell apoptosis by increasing the ratio of Bax/Bcl-2. Additionally, LEO increased the sensitivity of cells to cisplatin, probably by reducing multi-drug resistant associate protein expression, such as MRP1 and P-Gp (Lin et al., 2020).

#### 4.4.4 Anti-multiple myeloma

Multiple myeloma (MM) occurs in the bone marrow blood system, which can lead to kidney damage, hypercalcemia, bone destruction, and anemia caused by bone marrow failure (Cowan et al., 2022). It was found that LEO could significantly enhance the maturation and activity of monocyte-derived dendritic cells (mo DCs) from healthy donors (HDs) and MM patients to enhance the immune system’s ability to recognize and clear tumors. Specifically, LEO upgraded the expression of CD83, HLA-DR, and CD40 both in HDs and mo DCs with 1 μM for 8 days. In metabolomics studies, LEO was discovered to have a prominent regulatory effect on the arachidonic acid metabolic pathway. In this metabolic pathway, 16 metabolites were remarkably upregulated and 2 metabolites were noticeably downregulated in mo DCs treated with LEO. However, LEO adjusted the activity of DCs to strengthen their ability to kill cancer cells by affecting their metabolism (Chen et al., 2023).

### 4.5 Protective effect on orthopedic disorders

Osteoporosis (OP) and osteoarthritis (OA) are the most common orthopedic diseases in the elderly population. Although OP and OA are two different diseases, experimental and clinical researchers have found that inflammation, obesity, hormones, osteopontin, drugs, and other factors co-influence the development of the two diseases (Bultink and Lems, 2013; Bai et al., 2022). LEO affects the inflammatory process and the biological activity of chondrocytes, synoviocytes, osteoclasts, osteoblasts, and bone marrow mesenchymal stem cells (BMSCs).

#### 4.5.1 Anti-osteoporosis

OP is a systemic bone disease that is prone to fractures due to a series of reasons, such as decreased bone mineral density and bone mass, destruction of bone tissue microstructure, and increased bone fragility (Muñoz et al., 2020). Chondrocytes and osteoblasts derived from BMSCs are capable of secreting bone-derived growth factors and cytokines to model and remodel skeletal cells (Ponzetti and Rucci, 2021). Osteoclasts originate from hematopoietic stem cells that directly absorb minerals such as calcium and phosphorus in bone and convert them into bone morphogenetic proteins and a variety of biological factors that can be taken up (Park-Min and Lorenzo, 2022). Osteoblasts and osteoclasts coordinate and interact with each other to participate in the development and maintenance of bone size, shape, and integrity. LEO treatment of MC3T3E1 osteoblasts promoted cell differentiation and increased the density and activity of alkaline phosphatase (ALP) in a concentration-dependent manner within the 0.1–10 μM range. LEO also upregulated the osteogenic markers ALP, Runt-related transcription factor 2 (Runx2), and osteoblast-specific gene α1-1 collagen in mRNA and protein levels in MC3T3E1. In addition, WB results revealed that the protein contents of Runx2 and β-catenin in the LEO administration group were significantly higher than those in the control group, and the bone loss caused by estrogen deficiency was also ameliorated in ovariectomized mice (Yang et al., 2018). In addition, ROS, released by damaged mitochondria, and apoptosis factors stimulate the autophagy program in osteoblasts, leading to cell death or apoptosis, which destroys the balance of bone metabolism and causes bone metabolic disorders (Wang et al., 2020). LEO could protect against H_2_O_2_-induced oxidative damage in rat BMSCs and reduce intracellular ROS and the mRNA levels of reactive oxidation markers COX2 and NOX4 in a dose-dependent manner in the range of 2–200 μM, and the protective effect was the best when LEO concentrations reached 10 μM. Meanwhile, LEO restored impaired mitochondrial function, activated mitophagy, maintained mitochondrial membrane potential, and upregulated the ATP level. LEO also promoted the differentiation of BMSCs into osteoblasts, as indicated by the increased expression of osteogenesis-related markers at the mRNA level (osteopontin, osteopontin, and Runx2) and protein level (osteoprotegerin and Runx2) at a dosage of 10 μM (Zhao et al., 2021; Zhao et al., 2022).

#### 4.5.2 Anti-osteoarthritis

OA is a non-inflammatory degenerative joint disease characterized by synovial inflammation, progressive degeneration of cartilage and related ECM, osteophyte formation, and subchondral bone sclerosis. Lesions first occurred due to ECM degradation by matrix-degrading enzymes (including gathered enzymatic ADAMTS and matrix metalloproteinases MMPs), leading to cartilage component harm (Rim et al., 2020). Concurrently, cartilage injury induces the production of pro-inflammatory cytokines by synovial cells, such as IL-1β, IL-6, and TNF-a, which are elevated in synovial fluid, synovium, cartilage, and subchondral bone and further prompt ADAMTS and MMP secretion with activated synovial cells, causing cartilage destruction again (Motta et al., 2023). The expressions of matrix-degrading enzymes MMP-1, MMP-13, ADAMTS-4, and ADAMTS-5 were upregulated in rat chondrocytes induced by IL-1β, which could be significantly turned over by LEO pretreated 20 μM for 3 h. Simultaneously, IL-1β-induced chondrocyte apoptosis was also prevented, as indicated by the drop of caspase-1 activity in mRNA and protein levels of Bax and the increase of Bcl-2 in chondrocytes. LEO dose-dependently suppressed IL-1β-induced iNOS, NO, COX-2, PGE2, and many inflammatory cytokines expression at both mRNA and protein levels within the range of 5–20 μM. The OA rats, whose anterior cruciate ligament was transected (ACLT) in the knee joint, were treated with an intra-articular injection of 10 mM LEO after the surgery. Compared with the model group, the degradation of the cartilage matrix was effectively suppressed, concomitant with the inhibition of inflammatory responses and apoptosis (Hu et al., 2019; Hu et al., 2019; Chen et al., 2020).

### 4.6 Other effects

#### 4.6.1 Skin wound healing effects

Skin is the body’s immune barrier, and the dermis is exposed to all sorts of pathogen invasions, causing inflammation, infection, and adverse effects (Matejuk, 2018). Wound healing begins immediately after the appearance of skin damage and is initiated, guided, and maintained by a series of cytokines and growth factors involving local cells, the vascular system, and the extracellular matrix (Werner and Grose, 2003). LEO can promote angiogenesis and enhance the wound-healing process. Co-culture with HUVECs for 48 h showed that LEO promoted directional migration of endothelial cells in a dose-dependent manner (5–20 μM) and significantly increased the number of budding tubules. WB results demonstrated that the presence of LEO extremely increases the expression of p-ERK and p-mTOR, while deforolimus, an mTOR inhibitor, could reverse the effect. At the same time, two full-thickness wounds, each measuring 20 mm in diameter, were created on each side of the rats’ backs. Following this, the rats were administrated LEO at a dosage of 20 mg/kg for 28 days. The results indicated that the wound closure speed and healing rate of the treatment group were especially higher than those of the blank group, and the number of capillaries in the wound bed and edge was also expanded. In addition to encouraging angiogenesis, the process of collagen matrix deposition and remodeling was also accelerated in the wound after LEO treatment (Wang et al., 2018).

A flap is a tissue mass composed of skin and subcutaneous tissue with its blood supply. It is commonly used in facial plastic surgery, surgical wound repair, and soft tissue injury (Deramo and Rose, 2023). Ischemia-reperfusion injury caused by the obstruction of blood circulation is the main cause of flap necrosis. LEO can regulate oxidative stress, apoptosis, inflammation, and angiogenesis of random skin flaps and significantly improve the survival rate. Following the random flap transplantation, rats were given LEO for 7 days, including three doses (10, 20, and 30 mg/kg). There was a substantial increase in the expression levels of SOD1, HO-1, and VEGF and a decrease in the levels of caspase-1 and the ratio of Bax/Bcl-2 in the treatment group. The IL-6, IL-1β, and TNF-α levels were also inhibited. In addition, treatment with LEO led to a remarkable elevation in the phosphorylation levels of eNOS, Akt, and PI3K within the flap tissue. However, LY294002 (a broad-spectrum PI3K inhibitor) and L-NAME (a non-selective NOS inhibitor NG-nitro-L-arginine methyl ester) could counteract the protective effect of LEO on skin flaps. Therefore, LEO may promote skin flap survival through the activation of PI3K/Akt/eNOS (Chen et al., 2023). A multi-territory perforator flap is an individualized flap based on the inheritance and development of a traditional flap, which can reduce the morbidity of the donor site. Nevertheless, local flap necrosis is inevitable due to ischemia-reperfusion injury, lack of angiogenesis, insufficient vascular relaxation, and oxidative stress (Weinberg et al., 2022). Similarly, LEO (15 mg/kg/d for 7 days) predominately escalated perforator flap survival area and blood perfusions in rats. The mean vessel density and VEGF expression were augmented by LEO. Furthermore, it prevented the flap from oxidative stress or apoptosis, improving the perforator flap’s survival (Lin et al., 2020).

#### 4.6.2 Hepatoprotective effects

Apart from the aforementioned, LEO has noteworthy hepatoprotective activity, and related studies focus on alcoholic fatty liver disease, non-alcoholic hepatitis, iron-induced hepatotoxicity, and acute liver injury. Acute liver injury (AILI) caused by an overdose of acetaminophen (APAP) is the main predisposition to drug-specific liver disease. Excess APAP is metabolized by cytochrome P450 enzymes (CYPs) to produce toxic N-acetyl-p-benzoquinone imine (NAPQI), which is also a marker of hepatocyte death. After exceeding the detoxification capacity of GSH, NAPQI covalently binds to thiol groups in cellular proteins (especially mitochondrial proteins), leading to mitochondrial oxidative stress and dysfunction and ultimately resulting in hepatocyte necrosis (Yan et al., 2018). LEO could diminish the injury, apoptosis, oxidative stress, and inflammation of mouse primary hepatocytes (MPHs) induced by APAP. The serum levels of aspartate aminotransferase (AST) and alanine aminotransferase (ALT), the expressions of apoptotic proteins Bax and caspase-1, and the levels of oxidative factors ROS, MDA, and MPO, as well as the contents of inflammatory factors IL-6, TNF-α, and IL-1β, were significantly decreased in the AILI mice pretreated with 20 and 40 mg/kg LEO for 7 days, while the expressions of Bcl-2, GSH, GSH-Px, and t-SOD were markedly increased. LEO also caused inflammatory cell infiltration and hepatocyte necrosis in AILI mice. In addition, LEO increased the phosphorylation of PI3K, Akt1, and GSK3β in AILI mice but did not affect normal mice (Yu et al., 2023).

Alcoholic liver disease (ALD) is a liver disease originating from long-term or excessive alcohol consumption that brings out ethanol metabolism in the liver to induce apoptosis, endoplasmic reticulum stress, mitochondrial damage, or regulation of the liver inflammatory cell response, including steatosis, hepatitis, fibrosis/cirrhosis, and hepatocellular carcinoma. Currently, it is believed that oxidative damage and lipid peroxidation are some of the important pathogenesis mechanisms of ALD (Osna et al., 2017). As an antioxidant, LEO was able to inhibit alcohol-induced liver peroxidation injury with little cytotoxicity, as reflected by stimulating the activation of GSH and inhibiting the release of MDA in a dose-dependent manner (0–500 μM) in the human hepatic cell line LO2 (Wu et al., 2022).

Non-alcoholic fatty liver hepatitis (NASH), another metabolic fatty liver disease, has analogous pathological changes to ALD. Abnormal lipid metabolism in liver cells caused by obesity, insulin, or genetic factors leads to lipid accumulation. The accumulation of lipids makes hepatocytes more sensitive to injury, resulting in pathological changes such as hepatocyte death and the recruitment of inflammatory cells (Lee et al., 2022). LEO could prevent the pathogenesis of NASH: cellular lipid deposition and TG and TC concentrations in HepG2 and HL-7702 cells were effectively reduced by LEO (125, 250, and 500 μM). In mice with moderate steatohepatitis induced by MCD feeding, pre-administration of LEO at doses of 50, 100, and 200 mg/kg effectively mitigated the elevation of AST, ALT, TG, and TC. There was neither steatosis nor inflammation and fibrosis in the liver tissue. Further exploration unveiled significant changes in oxidative markers SOD, GSH, and MDA after LEO treatment, which repeatedly confirmed the anti-oxidative damage characteristics of LEO (Zhang et al., 2019).

As the main storage site of iron, the liver is extremely susceptible to iron poisoning due to iron overload. Characteristics of ferroptosis like iron metabolism, amino acid oxidation system imbalance, and lipid peroxide concentration were observed in a variety of chronic liver diseases and their different stages (Xiong et al., 2022). The iron content, the protein expression of lipid hydroperoxide and COX-2, and the activities of ALT and AST in the serum of iron-poisoning rats were surging, and the protein expression of GPx4, a competitive antioxidant enzyme for lipid peroxidation, was substantially restrained. LEO alleviated iron overload-mediated hepatotoxicity by acting on these ferritin biomarkers. Meanwhile, LEO (100 mg/kg/d for 10 days) attenuated iron overload-induced oxidative stress in rats’ liver tissue; it significantly upregulated GSH levels and the activities of GSR, GCL, GPx, CAT, and SOD and downregulated the levels of 8Oxo-dG and MDA. Additionally, LEO treatment inhibited the nuclear translocation of NF-κB and Nrf2 in liver tissues, with subsequent reductions in the protein levels of the downstream pro-inflammatory cytokines TNF-α and IL-1β and the antioxidant enzymes NQO1 and HO-1 (Salama et al., 2022).

#### 4.6.3 Renoprotective effect

Acute kidney injury (AKI) is a common clinical disease characterized by a rapid increase in serum creatinine, a decrease in urine output, or both. AKI often occurs as a complication of various syndromes, such as sepsis, cardiorenal syndrome, and urinary tract obstruction, which greatly adds obstacles to the treatment of AKI (Ronco et al., 2019). Cisplatin, a common anti-neoplastic drug, is mediated by local transporters such as OCT2 and MATE1 in the proximal renal tubules for uptake and excretion. The accumulation of cisplatin poisoning in proximal renal tubular cells leads to AKI. AKI is accompanied by oxidative stress, cell apoptosis and necrosis, local and systemic inflammatory responses, and dysregulation of autophagy (Holditch et al., 2019). LEO could resist oxidative stress injury, and preconditioning (7.5, 15, and 30 mg/kg/d for 7 days) was able to improve renal dysfunction and histological damage in rats with renal ischemia-reperfusion (I/R)-induced AKI, with the most significant effect in the 30 mg/kg dose group. Specifically, LEO promoted the expression of HO-1 and NQO-1, significantly increased the activities of SOD, CAT, and GSH, and decreased the level of MDA in I/R-induced AKI rats. Meanwhile, it was detected that the levels of IL-1β, TNF-α, IL-6, and IL-8 in serum increased after I/R, while LEO could remarkably convert inflammation. Further studies spotted that the protein levels of TLR4, MyD88, p-NF-κB, and p-IκBα decreased in I/R rats treated with LEO (Hu et al., 2022). In addition, another study demonstrated that ferroptosis was closely related to cisplatin-induced AKI (Hu et al., 2020). Ferroptosis is a novel form of regulatory cell death, that is, cell death caused by iron-dependent lipid peroxidation. LEO reversed RSL3-induced lipid peroxidation and ferroptosis in human renal tubular epithelial cells (HK-2) induced by cisplatin for 24 h. After the administration of 100 μM, LEO relieved the inhibition of HK-2 cell viability, upregulated intracellular GSH, and downregulated MDA and the biomarkers of ferroptosis, GPX4, and xCT. At the same time, the protein expressions of Nrf2, NQO1, and HO1 were also upregulated. Similar results were obtained *in vivo* experiments. Compared with the control group, cisplatin-induced severe renal tubular dilatation, renal tubular epithelial cell edema, cast formation, blood urea nitrogen, serum creatinine level, kidney injury molecule-1, NGAL, macrophage infiltration in renal tissue, and oxidative stress factor expression were especially inhibited by LEO (50 and 100 mg/kg). Furthermore, in the model group, cisplatin aggravated iron deposition in the kidney and increased membrane ferroportin receptor, ferritin heavy chain 1, and ferritin light chain protein and mRNA levels, which were restored by LEO in a dose-dependent manner (Hu et al., 2022).

## 5 Toxicology

*Leonurus* was listed as the top grade in Shennong’s Classic of Materia Medica, and it was considered to be non-toxic in each ancient herbal record. As one of the primary metabolites, understanding the toxicity profile of LEO is crucial for its clinical application and the prevention of adverse drug reactions. LEO is a central stimulant, with the effect of first excitement and then anesthesia on the CNS. It has similar muscle relaxant effects as tubocurarine and ergot sample contraction of uterine function, which can expand small arteries and decrease blood pressure. Specifically, it is manifested as a sudden feeling of general weakness, pain, numbness, or paraplegia. In serious cases, it is accompanied by sweating, hypotension, or even collapse, rapid and enhanced breathing, and in more severe cases, respiratory paralysis (Luo et al., 2020). LEO has no obvious tissue toxicity to experimental animals’ hearts, livers, and kidneys at therapeutic doses (10, 20, and 30 mg/kg). The impact of LEO treatment (10 mg/kg) for 178 days on the liver level of high-fat Rhesus monkeys was analyzed, and no marked change in serum ALT or aspartate AST between different groups during the experiment was observed. These suggest that it can be classified as a low-toxicity substance (Chen et al., 2023). Up to now, no scholars have carried out acute toxicity tests and long-term toxicity tests of LEO, but there are studies on the toxicity of total alkaloids in *Leonurus*. The acute toxicity test results showed that LD50 of 95% ethanol extract was 118.68 g/kg, and the viscera index of the liver and kidney, serum ALT, or AST, urea nitrogen (BUN), and creatinine (Cr) were also increased. This suggests the toxic target organs were mainly the kidney and liver but had no essence of pathological changes, and kidney toxicity was greater than that of the liver (Lv et al., 2015). Another subacute toxicity test showed that the total alkaloids of motherwort had a greater effect on the liver function of mice. The outcomes of the subacute toxicity test showed that the liver was more sensitive to the total alkaloids of *Leonurus* (Luo et al., 2010). The results of the long-term toxicity test revealed that after 90 days of high doses (30, 60, and 120 g/kg/d) of 95% ethanol extract, the levels of BUN and Cr in rat plasma increased, and dose-dependent renal injury appeared, which was manifested as mild inflammation and fibrosis in renal interstitium and mild tubular fatty degeneration (Lv et al., 2009). A long-term toxicity test was conducted to compare the degree of kidney injury between *Leonurus* water extract and 95% ethanol extract in rats, and it was discovered that both of them could induce kidney injury in rats after 9 weeks. The ethanol extract group was more obvious because the ethanol extract contained more alkaloids (Lu et al., 2019). The contents of total SH and MDA in rats’ blood in the ethanol extract group increased, and the level of GSH and the activities of SOD and GSH-Px decreased in a dose-dependent manner. This suggests that the renal injury caused by total alkaloids in *Leonurus* may be related to the enhancement of lipid peroxidation in renal cells induced by oxidative stress and also to the loss of active molecules-SH in tissues (Sun et al., 2014). At the same time, as mentioned above, LEO also has hepatorenal protective effects, which seem to be opposite to its liver and kidney toxicity. Generally, efficacy and toxicity are closely linked with dosage and time, and this needs more detailed research. LEO is often administered in gynecological and obstetrical diseases, so developmental and genotoxicity studies were carried out. The outcomes showed no significant maternal toxicity, embryonic toxicity, fetal toxicity, or teratogenicity at doses of 500, 1,000, and 2000 mg/kg (Y. Tian et al., 2020).

## 6 Clinic trail

Elevated blood lipids, or hyperlipidemia, pose serious risks of developing various CCVDs (Pérez de Isla, 2016). In pre-clinical experimental studies, three hyperlipidemia model tests confirmed that LEO’s total cholesterol and triglyceride lowering effects were superior to or close to statins, but the toxic reactions were much lower than statins (Suguro et al., 2018). Since 2018, several Phase I clinical trials of LEO for the treatment of hyperlipidemia have begun. These clinical trials conducted single or multiple administration studies on LEO’s tolerability, safety, pharmacokinetic profile, and effects of food on pharmacokinetics (illustrated in Table 3). Specific test results have not yet been published, but this is a giant step for LEO from bench to bedside.

**TABLE 3:** Clinical trial information of LEO.

Register number	Clinical trial subject	Drug	Indication	Trial stage	Trial condition	Applicant unit	Trial institution	Record date
CTR20221140	Phase Ib clinical trial of leonurine sulfate tablets	Leonurine sulfate tablets	Essential hyperlipidemia	In progress (recruiting)	Phase I	Zhuhai Hengqin New District Zhongzhu Zhengtai Medical Management Co., LTD./Fudan University	Shanxi Provincial People’s Hospital	2022/5/23
CTR20191574	Phase I continuous administration of leonurine sulfate tablets	Leonurine sulfate tablets	Hyperlipidemia	Completed	Phase I	Zhuhai Hengqin New District Zhongzhu Zhengtai Medical Management Co., LTD./Fudan University	Laboratory of Phase I clinical trial, Peking University First Hospital	2019/8/7
CTR20190238	Phase I clinical trial of dietary effects of leonurine sulfate tablets	Leonurine sulfate tablets	Hyperlipidemia	Completed	Phase I	Zhuhai Hengqin New District Zhongzhu Zhengtai Medical Management Co., LTD./Fudan University	Laboratory of Phase I clinical trial, Peking University First Hospital	2019/2/19
CTR20181929	Phase I clinical trial of leonurine sulfate tablets	Leonurine sulfate tablets	Hyperlipidemia	Completed	Phase I	Zhuhai Hengqin New District Zhongzhu Zhengtai Medical Management Co., LTD./Fudan University	Laboratory of Phase I clinical trial, Peking University First Hospital	2018/10/23
ChiCTR1800019071	Randomized, double-blind, tolerability, safety, and pharmacokinetic characteristics of leonurine sulfate tablets in healthy adult volunteers with a single administration	Leonurine sulfate tablets	Hyperlipidemia	Completed	Phase I	Zhuhai Hengqin New District Zhongzhu Zhengtai Medical Management Co., LTD./Fudan University	Peking University First Hospital	2018/10/25

## 7 Insight and perspective

In this paper, the extraction methods from herbs, synthetic pathways, biosynthetic mechanisms, pharmacokinetics, pharmacological effects in a variety of diseases, toxicology, and clinical trials of LEO are reviewed comprehensively. As a unique alkaloid of the genus *Leonurus*, LEO has shown good application prospects. At present, scholars at home and abroad have carried out profound research on the pharmacokinetics, pharmacological action, toxicology, and other aspects of LEO and have obtained a series of significant research results. At the same time, the biosynthesis and drug preparation technology of LEO have also made great progress, which provides important technical support for the industrial production of LEO. Fewer plant sources and a large demand force researchers to find more economical and efficient synthetic routes while optimizing the extraction process. However, as a drug, more detailed and in-depth research is needed from bench to bedside. The pharmacokinetic studies that have been reported so far have been conducted in experimental animals, and although there are some pharmacokinetic clinical trials underway, these alone are far from sufficient, and the problems of low bioavailability, high first-pass elimination, and hepato-enteric circulation may be addressed by structural modifications. LEO has shown a good therapeutic prospect for a variety of diseases, but the specific mechanism is not fully understood. It is also important that there are few studies on the toxicology and drug interactions of LEO. Safety evaluation and toxicological studies need to be reinforced to ensure its safety and effectiveness in clinical applications. Finally, it is supposed to intensify the clinical research of LEO, explore its application prospects in clinical treatment, and provide more reliable clinical evidence for its clinical application.

## Figures and Tables

**Table:** 

**LEO**	Leonurine
**DCC**	Dicyclohexylcarbodiimide
**HMPT**	Hexamethylphosphotriamide
**ADC**	Arginine decarboxylase
**SCPL**	Serine carboxypeptidase-like
**BRPP**	Plasma protein
**L-O-G**	Leonurine-10-O-β-D-glucuronide
**ESCs**	Endometrial stromal cells
**ER**	Aromatase-estrogen receptor
**PRB**	Progesterone receptor subtype B
**NK**	Natural killer cells
**HUVECs**	Human umbilical vein endothelial cells
**PPCI**	Primary percutaneous coronary intervention
**MIRI**	Myocardial ischemia-reperfusion injury
**ECM**	Extracellular matrix proteins
**a-SMA**	a-muscle actin
**MMP**	Matrix metalloproteinases
**TIMPs**	Tissue inhibitors of metalloproteinases
**TGF-β**	Transforming growth factor-β
**PDGF**	Platelet-derived growth factor
**Nox4**	NADPH oxidase 4
**IS**	Ischemic stroke
**OGD**	Photochemical
**VEGF**	vascular endothelial growth factor
**Aβ**	β amyloid protein
**NFT**	Neuronal fibrillary tangles
**HPA**	Hypothalamic-pituitary-adrenal
**GR**	Glucocorticoid receptors
**MR**	Mineralocorticoid receptors
**CORT**	Corticosterone
**OPCs**	Oligodendrocyte precursor cells
**SlC40A1**	Solute carrier family 40 member 1
**ALP**	Activity of alkaline phosphatase
**AST**	Aspartate aminotransferase
**ALT**	Alanine aminotransferase
**NASH**	Non-alcoholic fatty liver hepatitis
